# Histopathological Overview of Experimental Ulcer Models

**DOI:** 10.5152/eurasianjmed.2022.22312

**Published:** 2022-12-01

**Authors:** Erdem Toktay, Jale Selli

**Affiliations:** 1 Department of Histology and Embryology, Philosophy Doctor Degree, Faculty of Medicine, Kafkas University, Kars, Turkey; 2 Department of Histology and Embryology, Philosophy Doctor Degree, Faculty of Medicine, Alanya Alaaddin Keykubat University, Antalya, Turkey

**Keywords:** Histopathology,, experimental ulcer models,, indomethacin,, acetic acid,, pyloric ligation,, ethanol,, stress

## Abstract

Histopathology is the process of examining tissue that includes all the changes, when a diseased tissue shows compared to a healthy group with a result of a histological observation. Histopathology has become an essential process in medical experimental research and medical experimental models. Scientists have developed medical experimental animal models for these reasons and have pioneered new drug research for many years. One of these experimental researches is experimental ulcer models. This model, which was initially a single method, has led to the emergence of new models with the discovery of physiological processes on ulcers by scientists. Nowadays, researchers have performed many new peptic ulcer models on experimental animals over the years. The main point in the creation of the ulcer model is the increase in the stomach acid level and the removal or corruption of the gastric mucus. When the experimental models were examined histopathologically, it was seen that the most severe models were those induced by pyloric ligation, acetic acid application, and indomethacin. In these models, ulcer foci that progressed to the submucosa were common, while the superficial damage spreading to the entire surface was striking in the ethanol model. While epithelial losses are shown on the surface of the mucosa, foci of necrotic apoptotic cell clusters extending to the submucosa are shown according to the weight of the model. In addition, evidence of inflammation has been shared in almost all studies. All these results show that ulcer models can be created by many different mechanisms. However, similar findings were observed in almost all experiments. Whether the experimental model caused severe or mild ulceration changed the histological findings.

**Main Points**

- The discovery of new mechanisms in the formation of peptic ulcer and the research of new drugs for the treatment of ulcers have created experimental ulcer models.
- In all peptic ulcer models, it is aimed to increase the gastric acid level and reduce or remove gastric mucus.
- The clearest sign of peptic ulcer is ulcer foci and histopathological changes in the mucus.
- In all ulcer models, superficial or deep mucosal erosions occur, characterized by apoptosis and necrosis.
- The type of ulcer model determines the severity of histopathological damage.

## Introduction

Histopathology is the process of examining tissues that includes all the changes, when a diseased tissue shows compared to a healthy group with a result of a histological observation.^1^ It is accepted as the key finding in the diagnosis and treatment of the disease in medical pathology laboratories, especially in human tissues taken by surgical operations. In addition, it includes the whole of the methods used in the analysis of changes according to the healthy group in experimental studies.

Medical experimental research is an important process in the advancement of science. The data obtained in these studies provide the development of new disease pathways and treatment agents for diseases. Many different findings are evaluated in medical experimental research. This information can sometimes be biochemical and sometimes molecular. However, the information is usually numerical. Visual presentation of these findings is only possible with histopathology.^1,2^ In this respect, histopathology has become an essential process in medical experimental research and medical experimental models.

Since the beginning of humanity, stomach problems are one of the most important diseases that have existed. Among these problems, stomach ulcer is an important disease. There may be many different causes in its etiology.^3^ Therefore, its pathophysiology varies. There are many different cellular mechanisms in its pathophysiology, from swelling of cells to death.^4^ Different etiological causes create different pathophysiological processes. All these pathophysiological processes require good treatment. Incorrect and delayed treatment can lead to a process that can cause total loss of function in the stomach and even death, leading to the end of people's lives. Therefore, the treatment of this disease is an absolute necessity.

Today, many different agents are used in the treatment. However, the variability in the pathophysiology of the ulcer causes confusion in the preference for the active drug.^5^ Also, the possible side effects of existing drugs make long-term use of these drugs difficult and complicate effective treatment.^6^ For this reason, the development of new drugs with the least side effects and the most effective is a well-known goal for the scientific world. Scientists have developed medical experimental animal models for these reasons and have pioneered new drug research for many years. One of these experimental researches is experimental ulcer models. This model, which was initially a single method, has led to the emergence of new models with the discovery of physiological processes on ulcers by scientists. Nowadays, there are many ulcer models available. The models have been associated with stomach acid and gastric mucus in general.^7^

In medical experimental research, histopathology is the most frequently used finding in ulcer models. Indeed, examination of the disease in the tissue provides important information. In this review, the diversity and variability of histopathological data in different ulcer models were analyzed, and differences and similarities were discussed.

## Peptic Ulcer Etiology

Peptic ulcer (PU) has a multifactorial etiology and is a common chronic disease in adults. It is frequently seen in the antrum of the stomach and the proximal region of the duodenum in the digestive system.^8,9^ It can also be seen in the lower part of the esophagus, the anastomosis area created in gastric surgery, the ileum, the jejunum, and, rarely, in the heterotropic localization area of the gastric mucosa.^10^ The term PU refers to acid peptic injury of the digestive tract. These include histopathologically superficial defects/erosions involving the gastroduodenal mucosa, and the progression of the damage to the submucosal layer is defined as ulcer. The ulcer causes severe stomach pain, epigastric burning, and often leads to gastrointestinal bleeding. Most patients experience dyspeptic symptoms (bloating, indigestion, burning, and nausea). Most patients with uncomplicated PU can be successfully treated with a good early and accurate diagnosis.^11^ Unfortunately, bleeding and perforation, which are the most common complications of the disease, are seen in late and advanced cases and may even cause the death of the patients. Generally, PU disease is estimated to occur in approximately 5%-10% of the population, the incidence increases with age, and ulcer disease commonly occurs between the ages of 25 and 64 years.^6^ However, in the last 20-30 years, it has been reported that the incidence of PU disease has shown a decreasing trend worldwide, and epidemiological studies highlight that PU-related hospitalizations and deaths have decreased.^12,13^ Decreased data on the incidence of the disease may result from the development of new treatment protocols. Another reason for the decrease in the complications of PU disease may be related to the widespread use of anti-acid secretory drugs and the more rational use of nonsteroidal anti-inflammatory drugs (NSAIDs).^14-16^ Despite great advances in the research of the disease, the etiology of PU disease is not fully understood.

The main pathophysiological theory of PU formation is the disruption of the balance between aggressive factors (acid and pepsin secretion) and defense elements (secretion and action of mucus and bicarbonate) in the digestive system.^7,9^ The etiopathogenesis of gastric ulcer includes environmental factors such as stress, genetic factors, age, gender, physiopathological disorders, *Helicobacter pylori* (*H. pylori*) infection, alcohol use, misuse of NSAIDs, trauma, sepsis, hemorrhagic shock, and burn formation.^17,18^ All these reasons, sometimes alone and sometimes together, can cause PU.

## Peptic Ulcer Pathogenesis

Under normal conditions, there is a physiological balance between gastric acid secretion and gastroduodenal defense mechanisms. The main factor in the pathogenesis of PU is the deterioration of mucosal integrity and the balance between aggressive and defensive factors. In this abnormal pathogenesis process, abnormal increases in gastric acid and pepsin synthesis occur PUs with the help of infectious, environmental, and genetic factors.^19^ In other words, disruption of gastric mucosal integrity results in the formation of ulcerations in the wound area. Historically, the increased secretory capacity of stomach acidity with dietary factors and/or stress was thought to be the main cause of PU. However, the discovery of *H. pylori* infection and the increasing use of NSAIDs changed the hypotheses in the development of PU. Today, the use of NSAIDs and *H. pylori* infection seem to be the 2 main risk factors for PU. In addition, factors such as *H. pylori* and NSAIDs increase the secretion of mucous aggressive factors, they conduct more effects to impair defense mechanism^8^ and studies show that 89%-95% of cases develop due to *H. pylori* infection and NSAID using.^20^

It has been emphasized in the literature that 80% of duodenal ulcers and more than 60% of gastric ulcers are associated with *H. pylori*.^21^ While PU due to acid hypersecretion develops in 20% of chronically infected cases, gastric atrophy and intestinal metaplasia have been reported in some of the cases.^4^ Although *H. pylori* infection does not invade tissues, it causes severe inflammation in the tissue and associated immune response.^22^ The urease enzyme secreted by bacteria converts urea into ammonium chloride and monochloramine which toxic substances, and the toxic substances cause damage to epithelial cells.^23^ Proteases and phospholipases secreted by bacteria weaken the mucosal defense by breaking down the glycoprotein–lipid complexes in gastric mucus, and this event constitutes the first step in damage formation. As a result, *H. pylori* infection increases gastric acid secretion, which is one of the aggressive factors, and decreases bicarbonate production in the duodenum.^24^

In cases without *H. pylori* invasion, the main cause of gastric ulcers is the use of NSAIDs.^25^ Cases of NSAID-induced PUs are typically asymptomatic. While lesions such as subepithelial hemorrhages and erosions are found in more than half of people who use NSAIDs chronically, 15%-45% of the cases have asymptomatic ulcers are detected by endoscopic examination.^26,27^ Moreover, serious gastrointestinal system complications have been reported in 1%-4% of patients using NSAIDs continuously.^28-30^ The NSAIDs affect mucosal defense by inhibiting enzymes in prostaglandin synthesis and cause vascular endothelial damage, decreased blood flow, obstructive microthrombus formation, and activation of neutrophils. On the other hand, in stress-induced PU, PGs prevent neutrophil-induced mucosal damage and this information indicates that prostaglandins have an important defense mechanism against PU.^31^

Mucosal damage can also develop due to endogenous and exogenous active oxygen and free radical formation.^32^ Lipid peroxidation is generally a process that results in the interaction of hydroxyl radicals with the cell membrane and the formation of lipid-derived free radicals.^33^ Alcohol use also increases lipid peroxidation and triggers ulcer formation in the mucosa.^34^ It has been shown in rat studies that the use of pure ethanol causes acute gastric mucosal lesions.^35^ Ethanol breaks down the mucus layer that protects the stomach surface from acid and leaves the stomach surface vulnerable to hydrochloric acid. As a result, ulceration occurs on the stomach surface.

In shortly, many different etiological factor can cause ulcers. For a correct treatment protocol, these factors should be eliminated and the well-treatment protocol should be applied.

## Experimental Peptic Ulcer Models

It is known that PUs, which are common in society, are caused by many reasons such as personal differences, malnutrition and wrong drug use.^5^ Today, pharmacological drugs such as histamine H2-receptor antagonists, anticholinergic drugs, and proton pump inhibitors are used in the treatment of ulcers.^36-38^ In the pathophysiology of the disease, the presence of many factors together or alone causes the disease. This situation causes the therapeutic effect of existing drugs to vary from person to person.^6^ Moreover, many possible side effects of existing drugs require the investigation of new drugs and therapeutic agents. For this reason, researchers combined different chemical and physical conditions to induce the development of gastric ulcer seen in humans on rats.^7,39^ Thus, the pathophysiology of ulcer has been better understood and has facilitated the development of new drugs.

Researchers have performed many new PU models on experimental animals over the years. When the experimental models are examined, the main point in the creation of the model is the increase in the stomach acid level and the removal or corruption of the gastric mucus.^40^ In this respect, experimental models can be categorized as models related to gastric acid level and models related to gastric mucus. As a third group, the category of models that affect both ways can be added to this. Based on this information, we can classify ulcer models as follows.

1. Ulcer models associated with gastric acid level
  - Acetic acid-induced ulcer model
  - Pyloric ligation-induced ulcer model
2. Ulcer models associated with gastric mucus
  - Ethanol-induced ulcer model
3. Ulcer models associated with gastric acid and mucus
  - Stress-induced ulcer model
  - Non-steroidal anti-inflammatory-induced ulcer model

In this review study, the histopathological findings observed in commonly used models are discussed.

## Models of Ulcers Associated with Gastric Acid Level

**Acetic Acid-Induced Ulcer Model: **Indeed, researchers were able to induce gastric ulcers in mice by injecting acetic acid into the stomach with a vehicle.^41,42^ The most obvious difference of this model from other models is that it creates a chronic ulcerative effect on the gastric mucosa. Considering the length of the healing process of gastric ulcer, it makes it understandable that this model is preferred for chronic ulcer healing. With this model, it is a suitable method to test the efficacy of therapeutic agents on the healing process of chronic PUs to demonstrate the antisecretory and cytoprotective effects of new pharmacological agents.^43^

In the application of this model, rats are anesthetized after 24 hours of fasting. Then, the animals are administered diluted acetic acid (4%) (2 mL) with the aid of a flexible plastic catheter by entering 8 cm into the colon via the anus. It is then held upside down for about 2 minutes to prevent the acid from escaping. The experiment is terminated after 24 hours of acetic acid administration.^43,44^

Some studies on this model have been examined in the research. Kolgazi et al^45^ investigated the anti-inflammatory activity of the nestafin-1 molecule in the acetic acid-induced ulcer model. When histological findings were analyzed, it was determined that acetic acid was caused shedding in the surface epithelium, severe degeneration in the gastric glands, submucosal edema, and inflammatory cell infiltration in the mucosa and submucosa. In the treatment groups, the healing status was investigated based on the findings seen in the ulcer. In addition, the findings of the study were strengthened by immunohistochemistry. They investigated the mechanism of the effect via nuklear faktor kappa beta (NF-kB), COX-1, COX-2, prostacyclin 2 (PGI2), Prostaglandin 2 (PGE2) molecules at the tissue level.

In another acetic acid-induced study by Zhao et al.^46^ leukocyte infiltration and edema were observed in the gastric mucosa in the ulcer group. Necrotic cell tissue was observed in the surface cells. The treatment groups were analyzed according to the reduction of these findings. In the histopathological findings analyzed in the ulcer group of this study, dilatations in gastric gland cells, degenerated surface mucus cells, and ulcer pits on the stomach surface were observed in the mucosa. When the ulcerative areas were examined more closely, they saw signs of severe inflammatory cell infiltration and mucosal hemorrhage. In the treatment groups, the therapeutic effect was investigated by making comparisons according to this ulcer group.

In another study, the effectiveness of palmatine active ingredient in the acetic acid-induced model was investigated.^47^ In this study, ulcer superficial mucosal erosions and a few ulcer foci were observed. They saw an area characterized with inflammatory cells in the ulcer focal areas. On the other hand, they presented a decrease in the number of lesions and a recovery characterized by fewer inflammatory cells in the treatment group,

There are many similar studies in the literature. In almost all of these studies, findings were presented through spills and ulcer foci on the stomach surface. Researchers have extensively analyzed the treatment status of edema, inflammation, and ulcer foci. In histological evaluations, it was observed that the mechanism and level of damage were rarely investigated by immunohistochemistry.

**Pyloric Ligation-Induced Ulcer Model: **Another way of increasing stomach acid is the continual increase of gastric acid fluid as a result of the contraction of the sphincter between the stomach and the duodenum and the inability of the stomach acid to be neutralized by passing into the duodenum. This process, which often causes ulceration in people with gastric sensitivity, has led to the idea of developing an experimental model with surgical intervention. With this model, which is based on the binding of the pyloric part of the stomach, which is the connecting part of the stomach to the duodenum, ulceration is initiated at the pyloric end of the stomach.^48,49^ In this ulcer model, the picture can usually be severe. It is accepted as a severe ulcer model due to both starting this process with a surgical intervention and causing the gastric mucosa to digest itself. Although it is a difficult model, it may be a suitable model for testing new agents that inhibit acid secretion or neutralize gastric acid in pyloric occlusion processes.

When the researches about this model are examined, the gastroprotective effect of citalopram, an antidepressant drug, against ulcers due to stress and pyloric ligation has been investigated.^18^ When the histopathology findings of the study were examined, they observed ulceration in the mucosa, epithelial loss, and ruptured mucosal layer findings. In light of these findings, the level of improvement in the treatment groups was analyzed.

Wang et al^50^ tested the therapeutic agent in the pyloric binding ulcer model and in the ethanol-induced ulcer model in his study with a similar model.^50^ In their histopathological findings, widespread gastric glandular tissue loss, irregular glandular structure, and hemorrhage and submucosal edema formation were observed in the ulcer group. In the treatment groups, the level of healing was analyzed according to the ulcer group over these changes.

In another study conducted with this method, Al-Gabri et al^51^ showed that pyloric ligation causes the separation of mucosal cells, occlusion of blood vessels, and large submucosal edema with inflammation in the histological evaluation of gastric mucosa. In addition, scoring was used in this study to present the findings more strikingly.

The literature shows that this model has been less preferred in recent years. The reason for this may be the difficulties of the experimental model due to surgical intervention and the similar ulcer effect can be made more easily in other models with other models.

## Ulcer Models Associated with Gastric Mucus

**Ethanol-Induced Ulcer Model: **One of the causes of ulcer formation in society is excessive alcohol consumption. Indeed, scientists recognize ethanol as a risk factor for the development of gastric ulcers. The basis of this problem is that ethanol dissolves and breaks up the mucus that is firmly attached to the stomach lining. The gastric surface without mucus on its surface is exposed to gastric hydrochloric acid.^52^ Also, studies have shown that alcohol stimulates acid secretion. In addition, it causes some negative changes when ethanol passes from the intestinal mucosa to the blood fluid. It causes microvascular injuries by disrupting the endothelium surface of the vascular structures, especially in the gastric connective tissue. It has also been reported that ethanol is known to cause disruptions in the cellular oxidant–antioxidant balance. Impaired antioxidant production and increased oxidant levels initiate lipid peroxidation in the cell membrane and initiate cellular damage up to necrosis and apoptosis.^53,54^ In addition to all these, direct consumption of high percentage of alcohol leads to the formation of necrotic lesions with a toxic effect on the gastric mucosa.^55^

The most distinctive difference of this model, which distinguishes it from other models, is that the primary pathway in the formation of ulceration does not occur through a pathway associated with increased acid secretion. Therefore, while this model is not recommended for testing gastric acid secretion inhibitory agents, it is shown as a preferred ulcer model to demonstrate antioxidant activity and cytoprotective activity. In the formation of the experimental model, the rats are fasted for 24 hours and 5 mL/kg of absolute alcohol or 96% alcohol is given directly to the stomach with the help of gavage, and after 1 hour, the experiment is terminated and the stomach is taken and examined.^56,57^

When the researches about this model are examined, Mousa et al^58^ investigated the antiulcerogenic effect of *Cuphea ignea* extract against ethanol-induced gastric ulcer in rats. In their study, they showed multifocal edema and mononuclear infiltration of inflammatory cells in the submucosal region in the ulcer group. They found severe cellular losses in gastric mucosal cells and severe intervillous hemorrhages in the mucosa. The efficacy of therapeutic agents was compared according to the change in these findings.

We also conducted another study on this model and investigated the effect of persimmon fruit on the ethanol-induced ulcer model.^59^ We observed histologically that irregular gastric pits, necrotized epithelial and glandular cells in superficial mucosal cells and deep mucosa. In addition, lymphocyte cell increase was observed in the ulcer areas in the connective tissue. In this study, histopathologically, immunohistochemical evaluation was performed. Caspase-3 and NF-kB levels were evaluated immunohistochemically. In the treatment groups, the level of improvement in these findings was analyzed.

In another study by Tanyeli et al.^60^ the protective effect of salusin-α and salusin-β was tested in an ethanol-induced model. In the histology data of the study, hemorrhagic ulcer areas and tissue erosion on the stomach surface, high dilatation of cells in the mucosa, and congestion and hemorrhage in the connective tissue were detected in the ulcer group. Immunohistochemically, a high level of Caspase-3 positivity was observed in the ulcer group. When the treatment groups were examined, it was shown that the damage was reduced in the treatment group compared to the ulcer group.

This model has started to become a trending model in recent years. Researchers have started to prefer this model over other models.

## Ulcer Models Associated with Gastric Acid and Mucus

**Stress-Induced Ulcer Model: **Humans are exposed to many physical negative stimuli in the environment every day. Unfortunately, these negative physical stimuli come together in individuals and cause psychological reasons. This situation causes an increase in gastric acid due to stress or deterioration of mucus synthesis and causes ulceration.^61^

Rat models of this stress model have been developed so that researchers can better understand the pathophysiology of this type of ulcer and try new pharmacological agents for its treatment. In the formation of this model, restraint stress was initially applied. Subsequently, the model was strengthened by adding cold water retention and water immersion stresses.^62,63^ This synergistic acute model has been shown to cause stress-induced gastric lesions.^64^ When the pathophysiology of this model is examined, a mixed picture is seen, including an increase in acid secretion, a decrease in mucus production, and an increase in histamine synthesis, which triggers a decrease in the flow of vessels feeding the stomach.^65,66^

When the articles about this model are research, the protective effect of hydrogen in stress-induced gastric ulceration was shown in a study.^67^ In the histopathological findings of this study, hemorrhagic necrotic lesions of the gastric mucosa were reported in rats after 12 hours of stress. In addition, it was noted that Caspase-3 activity increased immunohistochemically in gastric mole cells in these regions.

In another study by Morsy et al.^68^ ulceration of the gastric mucosa due to cold restraint stress was shown. It has been mentioned that this situation causes a decrease in mucosal thickness, loss of surface epithelial cells, and irregularity in gastric glands. In addition, it is aimed to show the mechanism of ulcer formation and the efficacy of the therapeutic agent with COX-1 and COX-2 levels immunohistochemically.

Many different studies have been examined in the literature. It has been seen that the majority of these studies are publications of at least 15 years. In these studies, histopathological evaluation was performed in a few of them. In many studies, macroscopic stomach image was found to be sufficient as a finding. In recent years, this model is less preferred. This may be due to the fact that the degree of stress in experimental animals causes a large fluctuation within the animals and creates within-group differences.

**Nan-Steroidal Anti-Inflammatory-Induced Ulcer Model. **Anti-inflammatory drugs are one of the most commonly preferred drugs in cases of acute and chronic inflammation.^69^ In particular, drugs such as NSAIDs (aspirin, indomethacin, and ibuprofen) in this group are frequently consumed.^70^ Unfortunately, erroneous overdose of this drug produces undesirable side effects. Especially when this side effect has been shown to be related to the occurrence of gastric ulcer lesions, it has been revealed that these drugs should be used with caution. This widespread phenomenon, which is evidenced by patient profiles and scientific observations, has prompted the development of models of NSAID-induced gastric ulceration in rats.^71^ When the pathophysiology of the development of such ulcer lesions is evaluated, it includes disruption of gastric acid secretion and disruption of mucosal prostaglandin synthesis. In addition, the presence of NSAID-induced PUs commonly encountered in the community has made this model preferred as the most commonly used ulcer model.

Under normal conditions, the gastric mucosa stimulates bicarbonate ion and gastric protective mucus secretion with stimulating effects of bio-mediators called prostaglandins. This vital pathway ensures a continuous healthy blood supply of the gastric mucosa and protects the stomach from stomach acid by lining the inner wall of the released mucus.^72,73^ All mechanism that disrupts the synthesis and function of prostaglandins leads to gastric ulceration by affecting gastric acid and mucus secretion.^70,74^ Another negative effect of NSAIDs is the inhibition of the activities of cyclooxygenase enzymes (COX-1 and COX-2).^75-77^ When changes occur in the activities of these 2 enzymes, decrease in gastric mucosal blood flow, decrease in gastric bicarbonate and mucus secretion, and deterioration in platelet aggregation are observed.^78,79^ Epithelial cell damage, especially in the surface mucous cells, continues with decreased angiogenesis in the lamina propria. Subsequently, the migration of leukocyte cells to the connective tissue leads to the exacerbation of oxidative cell damage.^80^ In addition, the overproduction of reactive oxygen species in the tissue (production burst) leads to lipid peroxidation in the cell membrane, leading to the initiation of cellular damage. Academic studies confirm the oxidative stress–NSAID relationship in the occurrence of gastric mucosal damage.^79,81^

This model, which is frequently preferred in medical experimental studies, is based on the development of ulcer lesions within a period of 4-8 hours by administering ulcer-inducing NSAID agents to experimental animals at the end of the 24-hour fasting restriction (usually orally).^82,83^

When the researches about this model are examined, Eraslan et al^84^ demonstrated the efficacy of agomelatine in the experimental model of indomethacin-induced ulcer. When the histopathology of this study was examined in the ulcer group, epithelial loss and loss of integrity were observed in the gastric mucosa, while areas of hemorrhage and leukocyte infiltration were observed in the connective tissue. Hemorrhagic foci extending to the sub-mucosa were observed. However, wall damage in vascular structures was emphasized. Immunohistochemically, NF-kB immune positivity was associated with inflammation, and Caspase-3 positivity was associated with apoptosis in ulcer areas. The effect of therapeutic agomelatine was determined according to the damage findings observed in the ulcer group.

We also carried out a similar research paper.^85^ We demonstrated the effect of dragon fruit in an indomethazine-induced ulcer model. In this study, we show findings such as bleeding foci, edematous areas, disruption of gastric glands, and leukocyte infiltration in lamina propria in the ulcer group. In addition, in this study, we were able to obtain information about the mechanism of the damage by showing Caspase-3, Bcl-2-associated X protein (BAX), and NF-kB activities immunohistochemically. We analyzed dragon fruit extract in the treatment groups according to the change in these parameters.

In the study of Koc et al.^86^ the efficacy of oleuropein and thymol molecules was investigated in the indomethazine-induced ulcer model.^86^ When histological data were analyzed, remarkable ulcerative lesions were shown in the gastric mucosa in the ulcer group. Epithelial cell losses, congestion, hemorrhage, and cellular infiltration findings were emphasized in the lesion areas. In addition, amyloid deposits consisting of immunoglobulins accumulated in the tissue are shown by special staining. These deposits have been associated with ulceration.

Like the examples earlier, there are many different studies in the literature. The presence of many current studies in recent years shows that this model is still actively used. The reasons for the widespread use of this model may be that the experimental model can be easily induced, gastric ulceration shows a great correlation in animals within the group, and many mechanisms that occur in the ulcer reaction occur in this model.

## Conclusion

Stomach ulcer is still one of the most important problems today. There are many different mechanisms in the pathophysiology of ulcer, which increase the gastric acid level and reduce the mucus on the stomach surface. This pathophysiological process causes deep ulcerations on the stomach surface, leading to partial or total tissue loss. This long-lasting pathophysiological process may initiate a pathological process leading to loss of stomach function and even cancer. Therefore, treatment is an absolute necessity. Many different drugs are routinely used in the treatment. However, the side effects of existing drugs and individual differences in treatment necessitate medical experimental research for the development of new drugs and a better understanding of the disease. Different mechanisms known for ulcer formation have contributed to the diversification of ulcer models. In this respect, researchers have determined a model according to the predicted mechanism on the efficacy of therapeutic agents. Overall, experimental models have evolved to be effective in increasing gastric acid level and removing or reducing mucus from the stomach surface. In our review, we revealed the differences and similarities of histological data in different experimental models.

When the experimental models were examined histologically, it was seen that the most severe models were those induced by pyloric ligation, acetic acid application, and indomethacin. In these models, ulcer foci that progressed to the submucosa were common, while the superficial damage spreading to the entire surface was striking in the ethanol model. Histologically, mucosal damage was emphasized histologically in almost all studies. While epithelial losses are shown on the surface of the mucosa, foci of necrotic apoptotic cell clusters extending to the submucosa are shown according to the weight of the model. In addition, evidence of inflammation has been shared in almost all studies. Researchers have supported their histopathology with immunohistochemical staining showing inflammation and cell death in some studies.

All these results show that ulcer models can be created by many different mechanisms. However, similar findings were observed in almost all experiments. The severity of histopathological findings varies only according to the selected model. As can be understood, all these data seen in ulcer present a common finding pattern. This raises the idea of developing a common histopathology assessment chart for ulcer histopathology
